# Designing for to Designing With: Co-Designing Interventions With Dementia Care Partners

**DOI:** 10.1093/geroni/igaf122.810

**Published:** 2025-12-31

**Authors:** Nicole Werner

**Affiliations:** Vanderbilt University Medical Center, Nashville, Tennessee, United States

## Abstract

Co-design as applied to the design, evaluation, and implementation of interventions for family and friend care partners of people living with dementia has great potential to address the needs and outcomes that matter most to dementia care partners. Co-design democratizes the intervention pipeline through sustained partnership in which co-designers have the decisional and generative power to identify the problem to be solved and collaboratively create the solution. However, fully implementing intervention co-design with dementia care partners can be challenging given care partners’ context and the current structures of academic research. This talk will describe one researcher’s journey from ‘designing for’ to ‘designing with’ family and friend care partners of people living with dementia, sharing approaches, strategies, and lessons learned across ten years of research.

